# Serum phospholipid fatty acids, dietary patterns and type 2 diabetes among urban Ghanaians

**DOI:** 10.1186/s12937-017-0286-x

**Published:** 2017-10-02

**Authors:** Franziska Jannasch, George Bedu-Addo, Matthias B. Schulze, Frank P. Mockenhaupt, Ina Danquah

**Affiliations:** 1 Department of Molecular Epidemiology, German Institute of Human Nutrition Potsdam-Rehbruecke (DIfE), Arthur-Scheunert-Allee 114-116, 14558 Nuthetal, Germany; 2 Komfo Anokye Teaching Hospital, School of Medical Science, Kwame Nkrumah University of Science and Technology, Kumasi, Ghana; 3 Institute of Tropical Medicine and International Health, Charité – Universitaetsmedizin Berlin, corporate member of Freie Universitaet Berlin, Humboldt-Universitaet zu Berlin, and Berlin Institute of Health, Augustenburger Platz 1, 13353 Berlin, Germany; 4 Institute for Social Medicine, Epidemiology and Health Economics, Charité – Universitaetsmedizin Berlin, corporate member of Freie Universitaet Berlin, Humboldt-Universitaet zu Berlin, and Berlin Institute of Health, Campus Charité Mitte, 10098 Berlin, Germany

**Keywords:** Type 2 diabetes, Dietary patterns, Fatty acids, Cross-sectional analysis, Lipid metabolism

## Abstract

**Background:**

Previously, a “purchase” pattern (rich in vegetable oil, manufactured foods, red meat and poultry, fruits, and vegetables) was identified among adults in urban Ghana and was inversely associated with T2D, while a “traditional” pattern (rich in fish, palm oil, plantain, green-leafy vegetables, beans, garden egg, fermented maize products,) increased the odds of T2D. To investigate, if specific fatty acids (FAs), partly reflecting the intakes of certain food groups and cooking methods, might explain the observed diet-disease relationships, serum phospholipid fatty acid profiles were characterized and their relationships with blood lipids that are common risk factors for T2D were analyzed.

**Methods:**

The relative proportions of 28 FAs (%) in 653 Ghanaians without T2D were measured by gas chromatography. In a cross-sectional analysis, the associations of FAs with dietary patterns and with serum lipids that are likely involved in T2D development were investigated. The FAs distributions across dietary pattern scores were examined. Standardized beta coefficients (β) were calculated for the associations of dietary pattern scores (per 1 standard deviation (SD) increase) with FAs. Across the tertiles of selected diet-related FAs, adjusted means of serum triglycerides, cholesterol, HDL-cholesterol and LDL-cholesterol were calculated.

**Results:**

In this mainly female (76%), middle-aged (mean age: 46.4, SD: 15.3 years) and predominately overweight study population (mean body mass index: 25.8, SD: 5.4 kg/m^2^), saturated FAs (SFAs) contributed 52% to total serum FAs, n-6 polyunsaturated FAs (PUFAs) 27%, monounsaturated FAs 12%, n-3 PUFAs 9% and trans FAs (TFAs) <1%. The “purchase” pattern was related to lower proportions of n-3 PUFAs (β per 1 score SD: −0.25, *p* < 0.0001), but higher proportions of linoleic acid (LA) (β per 1 score SD: 0.24, p < 0.0001). The “traditional” pattern was characterized by lower proportions of arachidic acid (β per 1 score SD: −0.10, *p* = 0.001). LA was inversely associated with triglycerides, but positively with HDL-cholesterol and LDL-cholesterol.

**Conclusions:**

In this Ghanaian population, serum FA profiles reflected the intake of key components of dietary patterns, such as fish and vegetable oil. FAs from manufactured foods (SFAs) and deep-fried meals (TFAs) did not contribute to the observed associations between dietary patterns and T2D. Still, LA might partly explain the health-beneficial effect of the “purchase” pattern.

**Electronic supplementary material:**

The online version of this article (10.1186/s12937-017-0286-x) contains supplementary material, which is available to authorized users.

## Background

Diabetes mellitus is a global health challenge, affecting 415 million people worldwide [1], the majority being patients with type 2 diabetes (T2D). Next to the Middle-East, sub-Saharan Africa is facing the second-highest growth rates of T2D globally [1]. Already, the prevalence of T2D in urban Ghana (10%) equals the figure seen among the adult European population [2]. Population aging and rapid urbanization that contributes to dietary changes and reduced physical activity are among the causes for this development in sub-Saharan Africa [3].

Therefore, we previously evaluated the importance of dietary patterns for T2D among adults living in urban Ghana [4]. By means of Principal Component Analysis (PCA), two dietary patterns were identified that showed distinct T2D-associations. The “purchase” pattern was characterized by high factor loadings of sweets, rice, meat, fruits and vegetables and reduced the odds of T2D by 59% per 1 standard deviation (SD) increase of the pattern score. The “traditional” pattern was characterized by plantain, cassava, green leafy vegetables, fish, fermented maize products and palm oil and increased the odds of T2D by 56% [4]. The role of food groups was evaluated to understand these unexpected associations. Sweets and soft drinks were rarely consumed; types of meat comprised mainly goat, sheep and bush meat; and fruits and vegetable consumptions were frequent, probably contributing to the inverse effect of the “purchase” pattern. The preference of carbohydrate-dense, satiating staples may partly explain the direct association of the “traditional” pattern with T2D [4].

Additionally, specific nutrients and metabolites, including serum phospholipid fatty acids (FAs) might contribute to the observed relationships. They constitute an objective measurement of the intake of specific dietary fats and thus, are valuable instruments to examine diet-disease relationships [5]. For fish-derived FAs, moderate correlations (*r* = 0.13–0.71) with blood lipid fractions were seen [6], while some specific odd-chained saturated FAs (SFAs) partly represent the intake of dairy fat (*r* = 0.23–0.62). Trans FAs (TFAs) correlate with the consumption of refined oils (*r* = 0.29–0.63), but can also stem from dairy [6]. However, some FAs, including palmitic acid, stearic acid and n-6 polyunsaturated FAs (PUFAs) are rather unsuitable markers of dietary fat, because their concentrations can be influenced by endogenous metabolism [7].

On the background of economic transition and rapid urbanization in Ghana, we suspect that a change in traditional food preparation from steaming towards deep-frying occurred. Consequently, TFAs should be increased in serum phospholipids. The abundant use of palm oil will lead to higher serum SFAs with possible detrimental effects on intermediate biomarkers of T2D [8].

Therefore, the aim of this study was to investigate the contribution of serum phospholipid FAs to the observed associations between dietary patterns and T2D. Specifically, the objectives were to i) characterize the serum phospholipid FA profiles, ii) to analyze the associations between dietary patterns and selected FAs serving as markers for dietary fat intake, and iii) to investigate the relationships of these FAs with blood lipids that are common risk factors for T2D.

## Methods

### Study design and study population

The Kumasi Diabetes and Hypertension (KDH) study was conducted as a non-matched hospital-based case-control study in urban Ghana between August 2007 and June 2008 [9]. In brief, patients from the diabetes center and the hypertension clinic were recruited, and preliminary controls came from friends, neighbors and parishioners, outpatients and hospital staff.

**Fig. 1:**
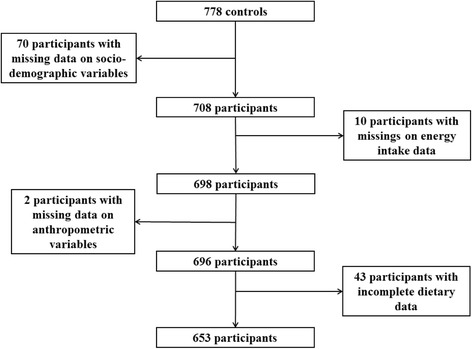
Flow diagram of excluded participants because of missing or implausible data

The study protocol conformed to the principles embodied in the Declaration of Helsinki and was reviewed and approved by the Ethics Committee of the School of Medical Sciences, University of Science and Technology, Kumasi. All participants gave informed written consent before they participated in the study. On the examination day, after 10-h over-night fast, venous blood samples were collected. Following breakfast, a personal interview was conducted on dietary intake, demographics, socio-economic status, medical history and lifestyle; and anthropometric measurements were taken. T2D was defined as fasting plasma glucose >7.0 mmol/L or as documented intake of anti-diabetic medication. Accordingly, there were 688 T2D cases and 778 controls without T2D. The latter were used for the present analysis, because diabetes status and anti-diabetic drugs can impact on FA metabolism [10]. Further 125 participants were excluded, because of missing data on dietary intake, demographics, socio-economics or anthropometrics. The final sample size was 653 (Fig. 1).

### Serum fatty acids and biochemical analyses

The proportions of the most commonly measured FAs in epidemiological studies [11] were analyzed in serum phospholipids. Fasting serum phospholipids reflect the dietary intake of FAs during the past weeks and are not influenced by recent FA intake [6]. The details of FA analysis are presented in Additional file 1. In brief, FAs were extracted from serum samples with tert-butyl methyl ether/methanol, followed by a solid phase separation, hydrolysis and methylation with trimethyl sulfonium hydroxide. The FA methyl esters were separated by their retention time in the gas chromatograph with a 100 m capillary column (HP-88) and detected by flame ionization. The 28 FAs were identified by standard substances and quantified as area percentage of each FA relative to the total area of all detected FAs.

Fasting glucose was measured in full venous blood in mmol/L by on-site photometry (Glucose 201^+^ Analyzer, HemoCue, Germany); inter-assay coefficients of variation ranged between 1.7% and 6.1%. Serum triglycerides and high-density lipoprotein (HDL)-cholesterol were measured by colorimetric assays (ABX Pentra400, Horiba Medical, Germany). The inter-assay coefficients of variation were 4.5% and 1.8%, respectively. Low-density lipoprotein (LDL)-cholesterol was calculated according to the Friedewald equation [12].

### Dietary assessment

In face-to-face interviews, trained study personnel applied a Ghana-specific food frequency questionnaire (FFQ) to capture the usual food intake of all participants over the last 12 months and to ensure, that the influence of daily and seasonal variation was minimized. The FFQ comprised 51 food items. According to their culinary use and similarities in their nutrient profile, these items were collapsed in the following 10 categories: starchy roots and tubers; cereals and cereal products; animal products; legumes, nuts and seeds; fruits; vegetables; fats and oils; salt and spices; sweets; and beverages (Additional file 2). The weekly intake frequencies were captured by six categories: never, seldom (<time per week), 1–2 times per week, 3–4 times per week, 5–6 times per week, and daily.

### Assessments of covariates

In personal interviews, we obtained demographic and socio-economic data: age, sex, education (none, primary, secondary, tertiary, other), literacy (not able, able with difficulties, able), occupation (subsistence farmer, commercial farmer, casual laborer, artisan, trader, business men, public servant, unemployed, other), presence of 11 household assets, and number of people living in the household. A socio-economic status (SES) sum score was constructed comprising the three major domains education, occupation and income. Details are explained in Additional file 3. Medical history included own and family history of diabetes and the use of lipid-lowering drugs. Smoking status and self-reported physical activity (work-related, transportation-related, leisure-time physical activity) were documented. Daily energy expenditure (kcal/day) was calculated as the sum of metabolic equivalents corresponding to activity intensity as metabolic equivalents (MET-hours) × body weight (kg) × duration (min).

Anthropometric data were obtained by trained personnel (all devices SECA, Germany). Weight (kg) was measured with a person scale, height (cm) with a stadiometer and waist circumference (cm) and hip circumference (cm) with a measuring tape. Body Mass Index (BMI) was calculated as weight/(height)^2^ (kg/m^2^) and waist-to-hip ratio (WHR) as waist circumference/hip circumference.

The common proxy markers education, occupation and income were used to construct a SES sum score ranging from 0 to 10 points. First, a new variable was constructed by combining the information on education and literacy. This new variable with four characteristics covered information about having formal education and being able to write and read; points from 0 to 3 were given. Occupation, originally a variable with nine characteristics, was condensed to a new variable with five characteristics, given the points 0 to 4. Due to differences in household structures and inflation rates of the local currency, income was assessed using a list of 11 household assets. An income score ranging from 0 to 12 points was constructed based on these assets and the number of people living in the household. The income score was divided into quartiles, given the points 0 to 3. To create the overall SES sum score, the points of education, occupation and the income score were summed up to a score ranging from 0 to 10 points.

### Statistical analysis

General characteristics of the study population and the proportions of single serum phospholipid FAs are presented for normally distributed metric variables as mean ± standard deviation, for non-normally distributed variables as median with interquartile range, and for categorical variables as percentage. Summarized proportions for the following major FA groups were calculated: SFAs, mono-unsaturated FAs (MUFAs), n-3 PUFAs, n-6 PUFAs, and TFAs. For comparisons between groups, Mann-Whitney-U test was applied for continuous variables and χ^2^-test was used for categorical variables.

Dietary patterns were constructed applying PCA for participants who had no T2D but FA measurements (*n* = 653), to evaluate the internal validity of previously identified dietary patterns. Details of the PCA analysis in the KDH study have previously been reported [4]. In brief, the 51 food items were collapsed into 33 food groups (Additional file 2) and were subjected to PCA using the PROC FACTOR procedure in SAS with an orthogonal rotation. The following criteria were applied to extract the optimal number of factors: eigenvalue >1, scree plot, and plausibility of the components. Standardized food intake weighted by factor loadings was summed to be able to rank the participants according to their adherence to each dietary pattern.

The distribution of general characteristics and the FA profile were examined across tertiles of the dietary pattern scores using χ^2^-test and trend test. For the associations of dietary patterns with FAs, Box-Cox-transformed FAs were calculated as median with interquartile range across pattern score tertiles. For those FAs that showed a significant trend across tertiles, linear regression models were fitted and adjusted for age, sex, family history of diabetes, SES sum score, energy intake, energy expenditure and WHR. For the associations of FAs with diabetes-related biomarkers, adjusted means and 95% confidence intervals (CI) of serum triglycerides, HDL-cholesterol and LDL-cholesterol were calculated across FA tertiles using the same set of adjustment variables. As a sensitivity analysis, multivariate linear regression models were calculated to analyze the associations of serum phospholipid FAs with fat-containing foods, characteristic of the respective dietary patterns.

## Results

### Study population

General characteristics of the study population are shown in Table 1. The majority was female, middle-aged and of low socio-economic status. One-fourth reported a family history of diabetes and less than 2% took lipid-lowering drugs. Smoking was prevalent in only 4% of all participants and largely restricted to men. Daily energy expenditure was 10% higher in men than in women. Mean BMI in women was higher than in men, while the WHR was lower. The concentrations of triglycerides were similar across gender, whereas LDL-cholesterol and HDL-cholesterol were significantly higher in women.

**Table 1:** General characteristics of the 653 Ghanaian participants

Characteristics	Total	Men	Women
n (%)	653	156 (23.9)	497 (76.1)
Age (years)	46.4 ± 15.3	46.0 ± 15.5	46.6 ± 15.3
FPG (mmol/l)	4.56 ± 0.71	4.58 ± 0.81	4.55 ± 0.67
Triglycerides (mmol/l)	1.18 (0.74)	1.12 (0.72)	1.19 (0.77)
LDL-cholesterol (mmol/l)	4.02 (1.69)	3.73 (1.99)	4.11 (1.62)^a^
HDL-cholesterol (mmol/l)	1.40 ± 0.39	1.30 ± 0.39	1.42 ± 0.39^a^
SES sum score	6.86 ± 2.38	7.02 ± 1.80	6.81 ± 2.54^a^
Daily energy intake (kcal/day)	1911 ± 657	2215 ± 678	1816 ± 621^a^
Daily energy expenditure (kcal/day)	1270 (776)	1372 (876)	1243 (779)^a^
BMI (kg/m^2^)	25.8 ± 5.36	23.3 ± 3.81	26.6 ± 5.52^a^
Waist:hip ratio	0.86 (0.10)	0.88 (0.10)	0.86 (0.10)^a^
Family history of diabetes (% yes)	25.3	19.9	27.0
Lipid-lowering drugs (% yes)	1.84	3.21	1.41
Smoking (% current or quit)	4.4	17.3	0.4^a^

Data were shown as means ± standard deviation for normally distributed metric variables, median (interquartile range) for metric variables without normal distribution and percentage of participants for categorical variables; ^a^ *p*-value ≤ 0.05 for significant differences between male and female participants

### Fatty acids profile

**Fig. 2:**
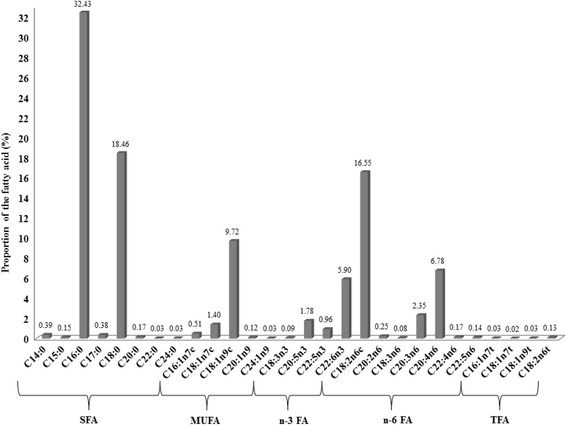
Proportions of 28 fatty acids in serum phospholipids of 653 urban Ghanaians

As presented in Fig. 2, SFAs contributed the highest proportion to total serum FA concentration with 52%, followed by PUFAs (36%), MUFAs (12%) and TFAs (<1%). In the group of SFAs, palmitic acid (16:0) and stearic acid (18:0) showed the highest proportions. In the group of MUFAs, oleic acid (18:1n-9) yielded the highest proportion, followed by vaccenic acid (18:1n-7). Eicosapentaenoic acid (20:5n-3) (EPA) and docosahexaenoic acid (22:6n-3) (DHA) made up almost 8%, and therefore highly contributed to n-3 PUFAs. The highest proportion in the group of n-6 PUFAs was observed for linoleic acid (18:2n-6) (LA), followed by arachidonic (20:4n-6) and dihomo-γ-linolenic acid (20:3n-6). Within the group of TFAs, the highest contribution was seen for linolelaidic acid (18:2n-6 t).

### Exploratory dietary patterns

By PCA, we identified virtually identical principal components as previously published (Table 2) [4]. The first component explained 14.5% and the second 8.0% of the total variance in food intake. Thus, sweets, soft drinks, hot chocolate, rice, margarine, vegetable oil, fruits and vegetables, meat, poultry, milk, eggs and plantain showed high factor loadings (≥ |0.30|) on the first pattern score. This component was labeled “purchase” pattern. For the second pattern score, palm oil, fish, cassava, fermented maize products, plantain, green-leafy vegetables, garden egg and beans yielded high factor loadings (AF 4). This component was labeled “traditional” pattern.

**Table 2:** Rotated factor loadings for the two dietary patterns in the Kumasi Diabetes and Hypertension Study

Food items	“purchase” pattern	“traditional” pattern
Sweets	**0.61**	−0.14
Juice	**0.60**	−0.16
Rice	**0.55**	0.06
Soft drinks	**0.52**	0.03
Lettuce	**0.52**	0.14
Carrot	**0.51**	0.13
Margarine	**0.50**	0.15
Milk	**0.50**	−0.06
Vegetable oil	**0.49**	−0.12
Fruits	**0.49**	**0.39**
Milo (chocolate drink)	**0.49**	0.03
Red meat	**0.48**	−0.07
Cucumber	**0.46**	0.09
Eggs	**0.45**	−0.06
Poultry	**0.39**	0.08
Groundnut	**0.33**	0.27
Porridge	**0.31**	0.06
Bread	0.27	0.26
Agushie (pumpkin seeds)	0.25	0.23
Coffee	0.22	0.10
Palm oil	0.17	**0.50**
Fish	−0.14	**0.49**
Green leafy vegetables	0.23	**0.48**
Cassava	−0.24	**0.48**
Banku (fermented maize product)	0.13	**0.47**
Plantain	**−0.33**	**0.46**
Garden egg (aubergine)	−0.01	**0.46**
Beans	0.22	**0.45**
Cocoyam	−0.12	**0.36**
Crab	0.03	**0.32**
Okra	0.24	**0.31**
Millet	−0.06	**0.30**
Yam	0.06	0.21

Food groups with factor loadings > 0.30, which are mainly characterizing the dietary pattern are captured in bold

Regarding the characteristics across the dietary patterns (Table 3), participants in the highest tertile compared to lower tertiles of the “purchase” pattern were significantly younger, had a higher SES, lower daily energy expenditure, lower WHR and nominally decreased fasting glucose. In the highest tertile of the “purchase” pattern, triglycerides were significantly lower than in the first two tertiles; the same trend was observed for LDL-cholesterol. For the “traditional” pattern, participants in the highest tertile compared to lower tertiles were significantly older, of lower SES, had higher daily energy intake, higher WHR, and nominally higher triglycerides and LDL-cholesterol (Table 3). For HDL-cholesterol and fasting glucose, no significant trends were observed.

**Table 3:** General characteristics of the 653 Ghanaian participants by tertiles of exploratory dietary patterns in 653 urban Ghanaians

Characteristics	“purchase” pattern score				“traditional” pattern score			
	Tertile 1	Tertile 2	Tertile 3	*p* for trend	Tertile 1	Tertile 2	Tertile 3	*p* for trend
N	218	217	218		219	213	221	
Age (years)	54.9 ± 13.2	47.7 ± 13.1	36.8 ± 14.0	<.0001	42.7 ± 16.4	47.3 ± 14.7	49.4 ± 14.2	<.0001
Sex (% male)	24.3	21.2	26.2	0.47	20.1	22.5	28.6	0.10
Diabetes family history (% yes)	24.3	25.4	26.2	0.91	29.7	23.0	23.2	0.19
Lipid-lowering drugs (% yes)	4.13	1.38	0	0.005	3.65	0.94	0.91	0.05
Prevalence of Hypertension	155	108	70	<.0001	88	123	122	0.0004
SES sum score	5.47 ± 2.58	7.20 ± 2.17	7.92 ± 1.59	<.0001	7.29 ± 2.23	6.75 ± 2.41	6.55 ± 2.45	0.002
Smoking (% current or quit)	6.9	2.8	3.7	0.09	3.6	4.7	5.0	0.77
Daily energy intake (kcal/d)	1934 ± 641	1898 ± 649	1902 ± 682	0.65	1756 ± 651	1883 ± 604	2097 ± 668	<.0001
Daily energy expenditure (kcal/d)	1342 (1104)	1289 (766)	1198 (632)	0.005	1237 (769)	1299 (716)	1267 (867)	0.67
Body Mass Index (kg/m^2^)	25.6 ± 5.22	26.6 ± 5.66	25.2 ± 5.09	0.23	25.6 ± 5.58	26.0 ± 5.28	25.8 ± 5.22	0.81
Waist-to-hip ratio	0.88 (0.10)	0.87 (0.08)	0.83 (0.11)	<.0001	0.85 (0.12)	0.87 (0.11)	0.87 (0.08)	0.0012
Fasting plasma glucose (mmol/l)	4.58 ± 0.70	4.57 ± 0.72	4.52 ± 0.71	0.39	4.51 ± 0.71	4.63 ± 0.77	4.52 ± 0.64	0.87
Triglycerides (mmol/l)	1.30 (0.91)	1.20 (0.64)	1.06 (0.63)	0.0001	1.07 (0.71)	1.21 (0.71)	1.24 (0.79)	0.06
HDL-cholesterol (mmol/l)	1.36 ± 0.43	1.42 ± 0.37	1.41 ± 0.37	0.19	1.41 ± 0.39	1.38 ± 0.39	1.40 ± 0.39	0.60
LDL-cholesterol (mmol/l)	4.19 (1.63)	3.97 (1.70)	3.87 (1.79)	0.10	3.97 (1.81)	3.96 (1.66)	4.11 (1.64)	0.99

Data were shown as means ± standard deviation for normally distributed metric variables, median (interquartile range) for metric variables for non-normal distribution and as percentage for categorical variables

*HDL* high-density lipoprotein, *LDL* low-density lipoprotein, *SES* socio-economic status

### Dietary patterns and fatty acids profile

The distributions of serum phospholipid FAs across the tertiles of the “purchase” and the “traditional” pattern scores are shown in Table 4.

**Table 4:** Distributions of 28 serum phospholipid fatty acids across tertiles of exploratory dietary patterns in 653 urban Ghanaians

Fatty acids (% of total fatty acids)	“purchase” pattern score				“traditional” pattern score			
	Tertile 1	Tertile 2	Tertile 3	*p* for trend	Tertile 1	Tertile 2	Tertile 3	*p* for trend
Saturated fatty acids	52.1 (2.68)	51.9 (2.78)	51.8 (2.15)	0.06	52.1 (2.61)	51.8 (2.63)	51.8 (2.54)	0.01
C14:0	0.39 (0.13)	0.39 (0.13)	0.39 (0.13)	0.61	0.38 (0.13)	0.40 (0.15)	0.39 (0.12)	0.90
C15:0	0.15 (0.05)	0.15 (0.05)	0.15 (0.05)	0.78	0.14 (0.05)	0.15 (0.06)	0.15 (0.04)	0.45
C16:0	32.3 (2.03)	32.4 (1.99)	32.5 (1.89)	0.76	32.5 (1.94)	32.3 (2.05)	32.4 (1.83)	0.44
C17:0	0.41 (0.14)	0.38 (0.11)	0.37 (0.10)	0.002	0.38 (0.13)	0.39 (0.13)	0.38 (0.10)	0.45
C18:0	18.6 (2.23)	18.3 (2.28)	18.5 (2.29)	0.34	18.7 (2.08)	18.2 (2.45)	18.4 (2.04)	0.15
C20:0	0.18 (0.09)	0.17 (0.08)	0.17 (0.07)	0.18	0.19 (0.09)	0.17 (0.08)	0.17 (0.06)	0.01
C22:0	0.03 (0.03)	0.03 (0.02)	0.03 (0.02)	0.90	0.02 (0.02)	0.03 (0.03)	0.03 (0.02)	0.07
C24:0	0.02 (0.01)	0.03 (0.01)	0.03 (0.01)	0.76	0.02 (0.01)	0.02 (0.01)	0.03 (0.01)	0.07
Mono-unsaturated fatty acids	12.1 (2.07)	12.0 (2.15)	11.6 (1.70)	0.61	11.7 (1.97)	11.9 (1.88)	12.0 (2.02)	0.14
C16:1n7c^a^	0.57 (0.33)	0.50 (0.32)	0.46 (0.29)	0.30	0.49 (0.33)	0.51 (0.32)	0.52 (0.31)	0.88
C18:1n9c^a^	9.83 (1.81)	9.77 (1.73)	9.63 (1.68)	0.70	9.59 (1.80)	9.67 (1.66)	9.88 (1.77)	0.25
C18:1n7c^a^	1.46 (0.46)	1.39 (0.43)	1.36 (0.39)	0.98	1.39 (0.50)	1.41 (0.38)	1.42 (0.42)	0.91
C20:1n9	0.12 (0.05)	0.13 (0.04)	0.13 (0.03)	0.92	0.12 (0.03)	0.13 (0.04)	0.12 (0.04)	0.97
C24:1n9	0.03 (0.03)	0.03 (0.02)	0.03 (0.02)	0.14	0.03 (0.02)	0.03 (0.02)	0.03 (0.02)	0.31
n-3 poly-unsaturated fatty acids	9.47 (3.36)	8.81 (2.76)	7.96 (3.03)	<.0001	8.36 (3.11)	8.75 (3.00)	9.09 (2.82)	0.50
C18:3n3	0.09 (0.05)	0.09 (0.04)	0.09 (0.03)	0.91	0.08 (0.03)	0.09 (0.05)	0.09 (0.04)	0.07
C20:5n3	2.01 (1.28)	1.80 (1.15)	1.55 (1.13)	0.01	1.65 (1.25)	1.90 (1.16)	1.83 (1.22)	0.93
C22:5n3	1.07 (0.41)	0.94 (0.39)	0.87 (0.38)	0.06	0.90 (0.43)	0.97 (0.43)	0.99 (0.37)	0.42
C22:6n3	6.40 (2.13)	5.93 (1.75)	5.22 (1.83)	0.001	5.73 (2.13)	5.83 (1.84)	6.05 (1.81)	0.50
n-6 poly-unsaturated fatty acids	25.4 (3.97)	26.7 (3.63)	28.0 (3.82)	<.0001	26.8 (3.78)	26.3 (4.95)	26.7 (3.46)	0.46
C18:2n6c^a^	14.8 (4.02)	16.3 (3.92)	18.1 (3.31)	0.001	16.8 (4.32)	16.1 (4.95)	16.5 (3.73)	0.82
C18:3n6	0.08 (0.04)	0.08 (0.05)	0.08 (0.04)	0.47	0.08 (0.04)	0.08 (0.04)	0.08 (0.05)	0.25
C20:2n6	0.26 (0.10)	0.26 (0.10)	0.25 (0.09)	0.92	0.24 (0.11)	0.25 (0.10)	0.27 (0.09)	0.02
C20:3n6	2.38 (1.04)	2.44 (0.89)	2.20 (0.93)	0.76	2.28 (1.01)	2.36 (1.00)	2.45 (0.94)	0.07
C20:4n6	6.83 (1.93)	6.75 (1.84)	6.74 (1.66)	0.76	6.69 (1.94)	6.76 (1.74)	6.88 (1.56)	0.50
C22:4n6	0.17 (0.08)	0.17 (0.09)	0.17 (0.08)	0.14	0.16 (0.08)	0.17 (0.08)	0.17 (0.08)	0.23
C22:5n6	0.15 (0.08)	0.14 (0.07)	0.14 (0.07)	0.98	0.14 (0.08)	0.14 (0.07)	0.15 (0.07)	0.45
Trans fatty acids	0.22 (0.09)	0.22 (0.06)	0.22 (0.07)	0.30	0.22 (0.08)	0.22 (0.07)	0.22 (0.06)	0.96
C16:1n7t^b^	0.03 (0.03)	0.03 (0.03)	0.03 (0.03)	0.98	0.02 (0.03)	0.03 (0.03)	0.03 (0.02)	0.25
C18:1n7t^b^	0.02 (0.02)	0.02 (0.02)	0.02 (0.02)	0.70	0.02 (0.02)	0.02 (0.02)	0.02 (0.02)	0.50
C18:1n9t^b^	0.03 (0.02)	0.03 (0.02)	0.03 (0.02)	0.84	0.03 (0.02)	0.03 (0.02)	0.03 (0.02)	0.23
C18:2n6t^b^	0.13 (0.05)	0.13 (0.05)	0.13 (0.05)	0.34	0.13 (0.06)	0.13 (0.05)	0.12 (0.05)	0.45

Data are shown as median (interquartile range). Fatty acids were Box-Cox-transformed and trend tests were corrected for multiple testing by False-Discovery-Rate

^a^cis-configuration

^b^trans-configuration

For the “purchase” pattern, there was no significant association with SFAs, except for lower proportions of heptadecanoic acid in higher tertiles, but this was not statistically significant after multiple adjustment for potential confounders (β per 1 score-SD increase: -0.06; SE: 0.05; *p* = 0.16). For MUFAs, lower proportions were observed for palmitoleic (16:1n-7), oleic and vaccenic acid across the “purchase” pattern tertiles. The n-3 PUFAs EPA and DHA, were significantly lower; this was still discernible after multiple adjustment (EPA [β: -0.24; SE: 0.05; *p* < 0.0001]; DHA [β: -0.25; SE: 0.04; *p* < 0.0001]). Within n-6 PUFAs, significantly higher proportions were observed for LA, which remained after adjustments (β: 0.24; SE: 0.04; *p* < 0.0001). While dihomo-γ-linolenic and arachidonic acid were lower across tertiles of the “purchase” pattern, no further n-6 PUFAs were significantly associated. In a sensitivity analysis, the positive association of the “purchase” pattern with LA was confirmed for the characteristic fat-containing foods margarine (β: 0.81; SE: 0.22; *p* < 0.0003), vegetable oil (β: 0.60; SE: 0.23; *p* < 0.01) and groundnut (β: 1.21; SE: 0.21; *p* < 0.0001).

Across tertiles of the “traditional” pattern, significant lower proportions of arachidic acid were discernible and remained after adjustment (β: -0.10; SE: 0.03; *p* = 0.001). EPA, docosapentaenoic acid (DPA), and DHA proportions were nominally higher across the tertiles of the “traditional” pattern. In the group of n-6 PUFAs, significantly higher proportions were observed for eicosadienoic acid, whereas proportions tended to be higher for arachidonic acid and lower for LA (Table 4). Also, in a sensitivity analysis, we verified the inverse association of the “traditional pattern” with LA for the characteristic fat-containing food items cassava (β:-0.95; SE: 0.22; *p* < 0.0001) and plantain (β: -1.09; SE: 0.22; *p* < 0.0001). After adjustment for respective confounders, no significant association remained for n-3 and n-6 PUFAs with the “traditional” pattern. Proportions of TFAs neither differed across tertiles of the “purchase” nor of the “traditional” pattern (Table 4).

### Fatty acids profile and blood lipids

For the associations of selected diet-related FAs with blood lipids, adjusted means of triglycerides, HDL-cholesterol and LDL-cholesterol across FA tertiles are presented in Table 5. For triglycerides, significantly lower concentrations across tertiles of eicosanoic acid, EPA and LA and higher concentrations across tertiles of DHA were observed. For HDL-cholesterol, positive associations were seen for EPA, DPA and LA. For LDL-cholesterol, lower concentrations across tertiles of the arachidic acid were observed, although not significant. Across the tertiles of the n-3 PUFAs and LA, the association with LDL-cholesterol was positive, however, not significant for EPA. These results were virtually identical after exclusion of participants on lipid-lowering drugs (2%).

**Table 5:** Distributions of serum lipid concentrations across tertiles of selected diet-related serum phospholipid fatty acids

Fatty acids (% of total fatty acids)	Serum lipids (mmol/L)	Fatty acid tertile 1	Fatty acid tertile 2	Fatty acid tertile 3	
		adjusted mean of serum lipids (95% CI)	adjusted mean of serum lipids (95% CI)	adjusted mean of serum lipids (95% CI)	*p* for trend
20:0 (median)		0.13	0.18	0.28	
Triglycerides		1.32 (1.25; 1.40)	1.16 (1.11; 1.22)	1.12 (1.05; 1.17)	0.0003
HDL-cholesterol		1.41 (1.36; 1.46)	1.41 (1.36; 1.46)	1.36 (1.31; 1.42)	0.20
LDL-cholesterol		4.14 (3.97; 4.32)	3.94 (3.78; 4.14)	3.71 (3.56; 3.90)	0.0003
20:5n-3 (median)		1.04	1.79	3.15	
Triglycerides		1.23 (1.17; 1.30)	1.23 (1.17; 1.30)	1.13 (1.06; 1.19)	0.01
HDL-cholesterol		1.35 (1.30; 1.40)	1.40 (1.35; 1.46)	1.44 (1.38; 1.49)	0.02
LDL-cholesterol		3.86 (3.67; 4.01)	3.90 (3.74; 4.10)	4.06 (3.86; 4.22)	0.12
22:6n-3 (median)		4.43	5.87	7.69	
Triglycerides		1.08 (1.03; 1.15)	1.20 (1.14; 1.26)	1.31 (1.25; 1.39)	<.0001
HDL-cholesterol		1.39 (1.33; 1.44)	1.40 (1.35; 1.45)	1.40 (1.34; 1.45)	0.79
LDL-cholesterol		3.78 (3.63; 3.97)	3.90 (3.74; 4.06)	4.10 (3.90; 4.26)	0.02
18:2n-6 (median)		13.1	16.5	19.9	
Triglycerides		1.31 (1.23; 1.38)	1.20 (1.13; 1.26)	1.09 (1.04;1.16)	<.0001
HDL-cholesterol		1.35 (1.30; 1.40)	1.38 (1.33; 1.43)	1.46 (1.40; 1.51)	0.01
LDL-cholesterol		3.74 (3.56; 3.90)	3.90 (3.74; 4.10)	4.14 (3.97; 4.35)	0.01

Means and 95% confidence intervals (CI) of serum lipid concentrations were adjusted for age, sex, diabetes family history, SES sum score, smoking, daily energy intake, daily energy expenditure and WHR

## Discussion

Previously reported associations of exploratory dietary patterns with T2D were partly unexpected and largely unexplained by key foods of the dietary patterns [4]. We hypothesized that food preparation and specific dietary fats might be responsible. Thus, we characterized the serum phospholipid FAs profile and investigated the relationships of FAs with dietary patterns and with selected intermediate biomarkers of T2D. In this urban Ghanaian study population, the FAs profile reflected key foods of the “purchase” pattern: lower n-3 PUFA proportions indicated lower fish intake, and higher LA possibly reflected vegetable oil intake. Overall, the proportions of SFAs and TFAs argue against a major role of food processing and adversely increased SFAs intake for the dietary pattern-diabetes relationships. Still, LA was associated with a favorable profile of serum lipids. This might explain some of the inverse association of the “purchase” pattern with T2D.

### Fatty acids profile

Lately, the recent epidemiologic literature covering the importance of circulating blood fatty acids has been reviewed [13]. Taken together, 13 observational studies with at least 100 healthy adults were published. The number of FAs under investigation differed greatly and focused largely on n-3 and n-6 PUFAs. None of them were from sub-Saharan Africa, but two studies described FAs among African-American individuals [14, 15]. More recently, Forouhi and colleagues added substantial findings from the European Prospective Investigation into Cancer and Nutrition (EPIC)-InterAct cohort and nine longitudinal studies among Caucasians for the relationships between PUFAs and T2D risk [16]. In comparison to Caucasians, the proportions were higher for SFAs, similar for MUFAs, higher for n-3 PUFAs, and lower for LA in the present study [13–16]. Still, comparability is constrained because FAs composition was measured in differential compartments, including adipose tissue [6], erythrocyte membranes [17] or serum free FAs [18].

Compared to African-American individuals, n-6 PUFAs appeared to be lower in the present study population [14, 15]. The differences between Caucasians and black US citizens are attributed to genetically determined activities of converting enzymes [15]. For n-3 PUFAs, the proportions were similar to those reported among blacks in a multi-ethnic cohort [14].

As to the few data from sub-Saharan Africa, proportions of serum phospholipid SFAs and MUFAs in the present analysis were similar to those in a small cross-sectional study among middle-aged Nigerians [19]. Compared to our study population, n-3 PUFAs (EPA 0.4% and DHA 3.1%) were remarkably lower in this indigenous population adhering to a dairy-based diet. TFA proportions were similar to those in our study [19]. Similar results for phospholipid FAs were seen in a study conducted among adolescent girls in Mozambique who rely on a maize- and rice-based diet [20]. Among Kenyan Massai [21], proportions of SFAs and n-3 PUFAs in lipids of red blood cells were lower than in the present study; MUFAs and n-6 PUFAs were higher.

### Dietary patterns, fatty acids profile and blood lipids

The “traditional” pattern, which was related to higher risk of T2D, was inversely associated with arachidic acid. In a European-wide study, arachidic acid was positively correlated with the intake of nuts and seeds, margarine, dairy products and poultry and inversely associated with T2D [22]. In fact, we and others observed an inverse association of arachidic acid with serum triglycerides [23]. Possibly, higher circulating concentrations of very long-chain FAs, including arachidic, behenic and lignoceric acid, compete with palmitic acid for the integration in respective ceramides and therefore might decelerate insulin resistance and β-cell dysfunction that are linked to ceramides with a high content of palmitic acid [24]. While the lack of association between arachidic acid and HDL-cholesterol in our study accords with recent findings [23], we could not confirm the positive association of arachidic acid with LDL-cholesterol [23]. The significant direct association of the “purchase” pattern and its fat-containing components with LA could possibly explain the inverse association of this pattern with T2D. We observed a more favorable lipid profile of lower triglycerides and higher HDL-cholesterol in the highest LA tertile. Indeed, in EPIC-InterAct, an inverse association of LA with T2D was discernible [16], possibly conveyed by cholesterol-lowering effects of LA-rich vegetable oils [25]. Yet, in our study population, LDL-cholesterol increased also across the tertiles of LA, arguing against a general cholesterol-lowering effect of LA. Moreover, controversy remains about the health impact of LA, which can also act as a precursor of pro-inflammatory metabolites and oxidized lipoproteins [26].

The “purchase” pattern was furthermore inversely associated with EPA and DHA proportions. Evidence for a risk reduction of T2D by dietary n-3 PUFAs are so far inconclusive [27]. Heterogeneous results may possibly stem from contaminated fish oil (supplements) [28]. In our study, EPA was positively associated with HDL-cholesterol and inversely with triglycerides, while results for LDL-cholesterol were not significant. DHA was positively associated with triglycerides and LDL-cholesterol, which is partly in line with observations from RCTs, suggesting a triglyceride-lowering effect [29, 30].

Only few studies investigated the role of FAs profiles for T2D among populations in sub-Saharan Africa. Thus, our findings contribute essentially to the knowledge in this field. Owing to the cross-sectional observational design of the present analysis, reverse causation might partly explain the deviation of our results from the findings in intervention studies. Nevertheless, the likelihood of reverse causation was minimized, because we have excluded participants with T2D and those who took lipid-lowering medication to account for diabetes-related changes of and interferences with lipid metabolism. We acknowledge that the metabolic profile of this specific study population might not be representative and hence, not generalizable to the Ghanaian population. Residual and unmeasured confounding may have distorted our findings, but regression models were adjusted for a large variety of relevant confounders. While the Ghana-specific FFQ has not been validated yet, it provides culture-specific dietary information.

FAs were expressed as percentages of total measured FAs from gas chromatography. Thus, we cannot interpret individual FAs independently of other FAs and imprecise estimates of FAs in very small proportions (e.g. TFAs) are likely [13]. Still, flame ionization detection enables to directly measure FA proportions and is a well-established measurement method. Other biological compartments with a slow turnover might better reflect the habitual fat intake, such as adipose tissue. However, the time frame of fat intake covered by the FFQ overlaps with the period reflected in fasting serum phospholipid fatty acids [6, 7]. Therefore, we consider the choice of the latter as a good compromise to investigate the associations between dietary intake and fatty acid composition.

## Conclusions

In conclusion, in this urban Ghanaian population, neither serum SFAs nor TFAs seemed to be responsible for the direct association of the “traditional” pattern with T2D. Alternative explanations for this relationship need to be explored. With regard to the “purchase” pattern, the association of LA with blood lipids was health-beneficial, possibly contributing to the inverse association of this pattern with T2D.

## Additional files

Additional file 1: Fatty acid (FA) analysis in serum phospholipids (PL) by gas chromatography (GC). (DOCX 16 kb)

Additional file 2: Overview of the 33 food groups and respective food items, that were collapsed into the food groups. (DOCX 19 kb)

Additional file 3: Construction of the socio-economic status (SES) sum score. (PNG 101 kb)

## Abbreviations

BMI: Body Mass Index
CI: Confidence interval
DHA: Docosahexaenoic acid
DPA: Docosapentaenoic acid
EPA: Eicosapentaenoic acid
FA: Fatty acid
HDL: High-density lipoprotein
LA: Linoleic acid
LDL: Low-density lipoprotein
MUFA: monounsaturated fatty acid
PUFA: Polyunsaturated fatty acid
SES: Socio-economic status
SFA: Saturated fatty acid
T2D: Type 2 diabetes
TFA: Trans fatty acid
WHR: Waist-to-hip ratio
