# Supplementary material for: Serum phospholipid fatty acids, dietary patterns and type 2 diabetes among urban Ghanaians
Source: Nutr J. 2017 Oct 2;16:63. doi: 10.1186/s12937-017-0286-x (PMC5625833; doi:10.1186/s12937-017-0286-x)
Supplement: Supplementary file 1 — Fatty acid (FA) analysis in serum phospholipids (PL) by gas chromatography (GC). (DOCX 16 kb) [file 12937_2017_286_MOESM1_ESM.docx]

**Additional file 1:** Fatty acid (FA) analysis in serum phospholipids (PL) by gas chromatography (GC)

Analysis of FA spectra of serum PL were performed with a strongly modified method using extraction with tert-butyl methyl ether/methanol, solid phase separation, hydrolysis and methylation with trimethyl sulfonium hydroxide (TMSH), and subsequent analysis by GC (Metges et al., 2003; Kaluzny et al., 1985; Baylin et al., 2005).

In detail, 25 µL of serum was transferred into 16 x 100 mm teflon coated screw capped vials. After addition of 1 mL dist. water lipids were extracted with 3 mL tert-butyl methyl ether (MTBE)/methanol solution (2/1, v/v) (MTBE containing 0.01% butyl hydroxytoluene (BHT)). The mixture was vortexed for 15 min at 720 min-1 using a rotatory shaker (KS 130 basic, IKA Werke GmbH & Co. KG, Staufen, Germany). After centrifugation (10 min, 2000 x g, 15°C) the upper layer containing the lipid fractions was transferred into another vial to evaporate under a stream of N2 at 40°C to dryness using a sample concentrator (SBHCONC/1, Dunn Labortechnik, Asbach, Germany) equipped with a block heater (SBH130D/3, Dunn Labortechnik). The dry lipid extracts can be stored at -20°C.

For bonded phase column separation (SPE) the dried lipids were redissolved into 500 µL chloroform. The mixture was applied to conditioned 1 mL SPE columns containing 100 mg aminopropyl-modified silica (Chromabond, MachereyNagel GmbH & Co. KG, Düren, Germany). The columns are placed on a vacuum elution apparatus equipped with vents and manometer (Carl Roth GmbH & Co. KG, Karlsruhe, Germany). Conditioning of columns was performed by washing with 2 x 1 mL n-hexane and 1 x 1 mL chloroform/i-propanol (2/1, v/v). The vacuum (~10 kPa) was released in time to prevent columns from becoming completely dry. Firstly, neutral lipids and free FA were eluted with 4 x 1 mL chloroform/methanol/acetic acid (100/2/2, v/v). After changing vials the PL were eluted with 2 x 1 mL methanol. Solvents were evaporated under a stream of N2 at 40°C and the dried PL can be kept at -20 °C.

For hydrolysis and methylation of FA the dried PL were redissolved in 200 µL toluene, vortexed, and transferred into GC-vials (32 x 11.60 x 0.90, IVA Analysentechnik e. K., Meerbusch, Germany). Ten µL of trimethyl sulfonium hydroxide solution (TMSH, 0.2 mol/L in methanol, Macherey-Nagel, 701 520.101) were added to form FA methyl esters (FAME) from FA of PL (except of sphingolipids). Vials were vortexed (30 min, 750 min-1, 40°C) using a thermomixer equipped with a block thermostate (MHR 23, DITABIS AG, Pforzheim, Germany). Samples containing the FAMEs were analyzed by GC. If necessary the samples can be stored at -20°C until analysis. Further, an internal standard (e.g. C21:0, heneicosanoic acid) can be added before TMSH reaction.

Analysis of FAMEs was performed using an Agilent GC system 7890A equipped with Agilent 7000 GC/MS Triple Quad (Agilent Technologies, Waldbronn, Germany) and a flame ionization detector (FID). The autosampler (MPS 2xL, Gerstel, Mühlheim an der Ruhr) was equipped with PAL stack and tray cooler system (CTC Analytics AG, Zwingen, Switzerland). 1 µL of sample was injected using a CIS 4C PTV-type GC-inlet (Gerstel) in splitless mode at 30°C, 30-260°C, ramp 12°C/s, held 2 min, 260-320°C, ramp 12°C/s.

The FAMEs were separated on a GC capillary column (HP-88, 100 m x 0.25 mm I.D., 0.2 µm film thickness, Agilent) using a constant He carrier gas flow of 1 mL/min. A GC oven temperature program was employed as follows: Start at 80°C, held 1 min, 80-170°C, ramp 10°C/min, held 5 min, 170-175°C, ramp 5°C/min, 175-190°C, ramp 2°C/min, 190-205°C, ramp 5°C/min, 205-235°C, ramp 5°C/min, held 8 min, total run time 57.5 min. The eluting gas flow was splitted for FID and MS detection (1:1) using a 2-way splitter and uncoated fused silica capillaries (Agilent) for restriction and adjustment of retention times.

For identification and column check-out a 37-component FAME mixture (SupelcoTM) was used. The mixture is available as a 100-mg neat mixture, containing C4–C24 FAMEs (2%–4% relative concentration).

FA composition of serum PL was expressed as area percentage of each FA relative to total area of all detected FA. The interassay coefficients of variation (CV%, n=10) for each serum FA using FID detection were: C14:0, 4.1; C15:0, 6.2; C16:0, 0.9; C16:1n7c, 3.7; C17:0, 1.9; C18:0, 1.1; C18:1n9c, 0.7; C18:1n7c, 1.9; C18:2n6c, 0.5; C20:0, 5.8; C18:3n3, 1.7; C20:1n9, 3.7; C20:2n6, 6.4; C20:3n6, 1.4; C20:4n6, 1.4; C20:5n3, 2.1; C24:0, 4.5; C22:4n6, 5.4; C22:5n63.1; C22:5n3, 3.4; C22:6n3, 3.1.

References

1. Metges CC, Lehmann L, Boef S, Petzke KJ, Müller A, Rickert R, Franke W, Steinhart W, Nürnberg G, Klaus S, cis-9,trans-11 and trans-10,cis-12 CLA affect lipid metabolism differently in primary white and brown adipocytes of Djungarian hamsters. Lipids 2003, 38:1133-1142.

2. Kaluzny MA, Duncan LA, Merritt MV, Epps DE. Rapid separation of lipid classes in high yield and purity using bonded phase columns. J Lipid Res 1985, 26:135-140.

3. Baylin A, Kim MK, Donovan-Palmer A, Siles X, Dougherty L, Tocco P, Campos H. Fasting whole blood as a biomarker of essential fatty acid intake in epidemiologic studies: Comparison with adipose tissue and plasma. Am J Epidemiol 2005, 162:373-381.
