# Supplementary material for: Serum phospholipid fatty acids, dietary patterns and type 2 diabetes among urban Ghanaians
Source: Nutr J. 2017 Oct 2;16:63. doi: 10.1186/s12937-017-0286-x (PMC5625833; doi:10.1186/s12937-017-0286-x)
Supplement: Supplementary file 2 — Overview of the 33 food groups and respective food items, that were collapsed into the food groups. (DOCX 19 kb) [file 12937_2017_286_MOESM2_ESM.docx]

Overview of the 33 food groups and respective food items, that were collapsed into the food groups

| Original food group | Original food item | Input variable of the factor analysis | Scientific rationale |
| --- | --- | --- | --- |
| Starchy roots and tubers | Cassava | Cassava |  |
|  | Plantain | Plantain |  |
|  | Cocoyam | Cocoyam |  |
|  | Yam | Yam |  |
|  | Sweet potato | - | Excluded, because 86% of the participants never consumed this item |
| Cereal and cereal products | Maize (Banku) | Maize (Banku) |  |
|  | Millet | Millet |  |
|  | Oats (Porridge) | Oats (Porridge) |  |
|  | Rice | Rice |  |
|  | Bread | Bread |  |
| Animal products | Fish | Fish |  |
|  | Red meat | Red meat |  |
|  | Poultry | Poultry |  |
|  | Eggs | Eggs |  |
|  | Milk | Milk |  |
|  | Crab | Crab |  |
| Legumes, nuts and seeds | Beans | Beans |  |
|  | Groundnut | Groundnut |  |
|  | Agushie (pumpkin seeds) | Agushie (pumpkin seeds) |  |
| Fruits | Orange |  |  |
|  | Mango |  | Single fruit items were combined into one food group “fruits” to account for dietetic similarity |
|  | Papaya | Fruits |  |
|  | Pineapple |  |  |
|  | Banana |  |  |
|  | Pae (avocado) |  |  |
| Vegetables | Tomatoes | - | Excluded, because 100% of the participants daily consumed this item and thus did not contribute to variation in the usual diet. |
|  | Pepper | - | Excluded, because 100% of the participants daily consumed this item and thus did not contribute to variation in the usual diet. |
|  | Garden egg | Garden egg |  |
|  | Okra | Okra |  |
|  | Green leafy vegetables | Green leafy vegetables |  |
|  | Carrot | Carrot |  |
|  | Cucumber | Cucumber |  |
|  | Lettuce | Lettuce |  |
| Fats and oils | Palm oil | Palm oil |  |
|  | Vegetable oil | Vegetable oil |  |
|  | Margarine | Margarine |  |
| Salt and spices | Salt | - | Excluded, because these items did not contribute to energy and macronutrient intake |
|  | Salt with iodine | - |  |
|  | Red pepper (dried) | - |  |
|  | Sugar | - |  |
| Sweets | Chocolate |  | Single sweets were combined in one group “Sweets”, because 89% of the participants consumed these item less than once a week |
|  | Ice cream | Sweets |  |
|  | Toffee |  |  |
| Beverages | Water | - | Excluded, because 100% of the participants daily consumed this item and thus did not contribute to variation in the usual diet. |
|  | Juice | Juice |  |
|  | Soft drinks | Soft drinks |  |
|  | Coffee | Coffee |  |
|  | Milo (chocolate drink) | Milo (chocolate drink) |  |
|  | Beer | - | Excluded, because >90% of the participants never consumed these items |
|  | Wine | - |  |
|  | Spirits | - |  |
