# Supplementary figures and images for: Serum phospholipid fatty acids, dietary patterns and type 2 diabetes among urban Ghanaians
Source: Nutr J. 2017 Oct 2;16:63. doi: 10.1186/s12937-017-0286-x (PMC5625833; doi:10.1186/s12937-017-0286-x)

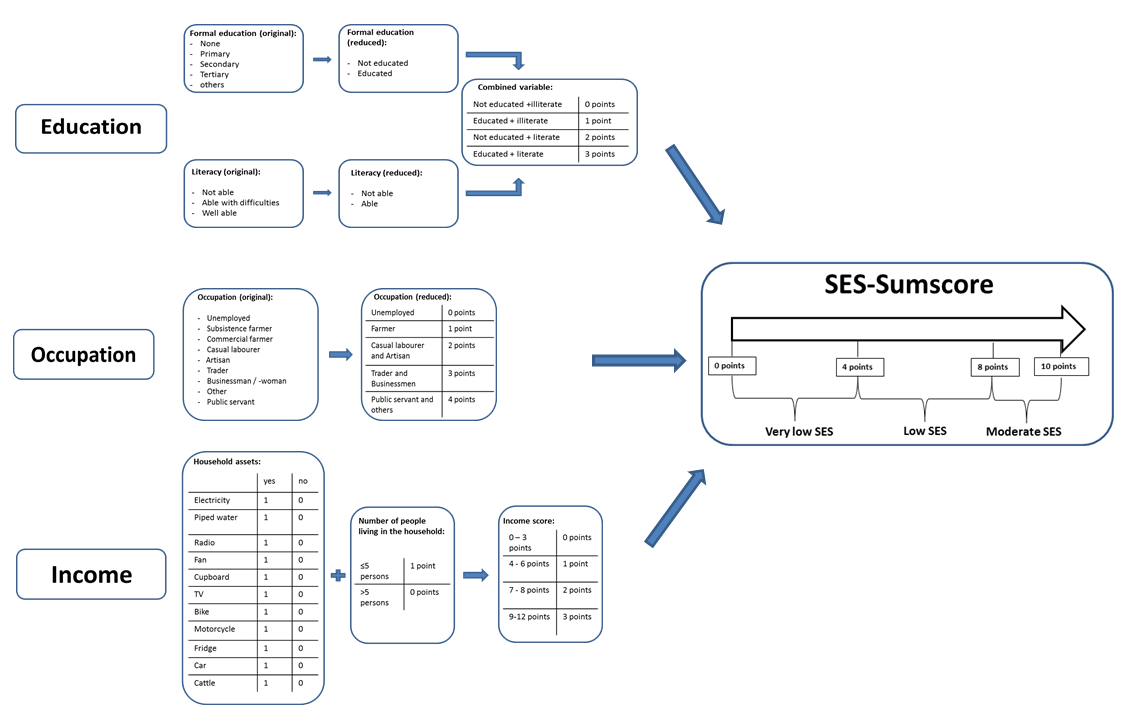

Supplement: Supplementary file 3 — Construction of the socio-economic status (SES) sum score. (PNG 101 kb) [file 12937_2017_286_MOESM3_ESM.png]
